# Medical Opinion and Movement

**Published:** 1908-11-07

**Authors:** 


### Medical Opinion and Movement.

At a recent meeting of the Ophthalmological Society of Copenhagen Hoeg read a paper on the use of massage in the treatment of cases of embolism of the arteria retinæ centralis, which gave rise to an interesting discussion. An abstract of the cases brought before the society is now published in the current number of the *Hospitalstidende.* Four patients with the typical symptoms were treated, and in each case the results were most encouraging. The average length of the treatment was four days, and the average age of the patients 52 years. The author gives details of his method, which consists in light rubbing movements, performed by the practitioner himself, over the eye-ball. The importance of accurate diagnosis before commencing the treatment was fully brought out in the discussion, and also the value of short sittings. Hoeg himself limits the treatment to ten minutes' daily massage, and keeps the patients on a low diet. Speakers following the author agreed that the massage treatment was exceedingly useful in selected cases, but pointed out that it was not suitable in all cases, and that it should be controlled by a systematic ophthalmoscopic examination before and after each sitting.

There are few subjects that of late years have aroused so much discussion in the profession as the origin of gall-stones. It is a question in which physicians and surgeons are both, and almost equally, interested, and the main contributions to the discussion have been made by both sections. Among the physicians Naunyn stands prominent as the author of the catarrhal theory, which supposes that the affection is due to a "lithogenous catarrh of the gall-bladder." According to this theory, the starting-point of all biliary calculi is the gall-bladder. This sac is affected with a catarrh, and the masses of mucus thrown out are the organic foundation upon which the bile salts are deposited. The theory and its objections are discussed in a recent number of the *Archiv fur Klinische Chirurgie*, where an interesting résumé of modern opinions on the pathogenesis of gall-stones is given. Thudicium, for example, scouts Naunyn's theory, and thinks that decomposition of the bile and the consequent precipitation of cholesterin are quite sufficient to explain the formation of calculi. Kramer, again, experimentally proved that the presence of bacilli was sufficient, in some cases at least, to lead to the formation of gall-stones, and his subsequent researches into the action of B. coli and B. typhosus as precursors of bile decomposition strongly supported the decomposition precipitation theory. More recently Litchtwitz has brought forward the colloidal theory which tries to explain the matter on purely physico-chemical grounds, and his report on the question has induced Exner and Heyrovsky, in the article above cited, to give a full account of their recent experiments. These appear fully to corroborate the earlier findings of Kramer and to put bacterial action in the forefront as the main factor responsible for calculi formation. They think that the bacillus of enteric fever plays a very prominent part in the pathogenesis of biliary calculi, and that it is the most important member of the bacterial flow of the gall-bladder.

The open operation for congenital dislocation of the hip is now confined to a few exceptional cases, and the majority of orthopædic surgeons agree that for all ordinary cases the "bloodless method," which has been popularised by Paci and Lorenz, is admirably suited. There is, however, still a want of unanimity, among English practitioners at least, upon the question of when and how this bloodless reduction should be carried out. Ostensibly Lorenz's method is used, but in many cases the treatment adopted differs very widely from that which is routine at Vienna. There has in consequence been a somewhat deplorable variety in the results obtained, and it seemed possible even at one time that the bloodless method might lose in popularity. The different and indifferent successes which have been obtained are due solely to want of thorough acquaintance with the technique of the method, a point which orthopædists would do well to insist upon. Mr. Barnes, in a recent number of the *British Journal of Children's Diseases*, contributes a clear and lucidly written paper on the treatment of congenital hip luxation. He is inclined to fix five or six years as the age-limit when the bloodless method is employed. While there can be no question that the ideal age is much below that estimate—Lorenz himself putting it as "within the first year"—there can be as little doubt that excellent successes have been obtained in cases of much older children. The essentials in the treatment are patience and perseverance, and the difficulties on which Mr. Barnes lays stress are by no means insurmountable. The paper will well repay perusal by the general practitioner, who should be better acquainted with the Lorenz method and its practical application than is at present the case.

Kohler gives an account, in a recent number of the *Munchener Medizinische Wochenschrift*, of three cases of obscure pains in the bones of the foot in children. He is disposed to regard the condition as a new disease, at any rate as a distinct and hitherto undescribed clinical entity. The main points in all the cases are the localisation of the pains, which appear to have been strictly limited to the dorsum of the foot, and the extraordinary changes of the small bones of the feet as revealed by repeated skiagraphic examinations. The *x*-ray photographs show the scaphoid bones much altered in shape, denser than normal, and apparently enlarged. The children limped and complained of tenderness along the inner border of the foot when pressure was made over the bones. No evidence of tubercle, syphilis, rickets, or osteomyelitis could be obtained in any one of the three cases, and the author admits that he cannot satisfactorily explain the condition in the light of our present knowledge. At the same time it must be pointed out that a tender condition of the feet, associated with slight planus and limping, is by no means uncommon in children of a school-going age, and doubtless many practitioners have met with somewhat similar cases. The $x$-ray revelations are interesting, but they are not sufficient by themselves to prove that the condition is entitled to rank as a new disease. It is satisfactory to learn that in every case the condition disappeared, and that three years later nothing abnormal could be found on skiagraphing the foot of one of the patients who, when first seen, had presented marked alterations in the contour and density of the scaphoid and semilunar bones of the feet. It would be interesting to find out whether the condition described by the author is a common one or not, for its bearings upon more important lesions of the foot seem obvious.

At a recent meeting of the Académie de Médecine M. Le Gendre presented a study of the factors (hygiene, profession, social position, mode of life, etc.) capable of modifying the symptomatology of gout. He distinguishes three clinical varieties of the disease, basing his classification upon a study of its articular manifestations. The first he calls the digestive variety, characterised by gastro-intestinal, pancreatic and hepatic disturbances. The second or angio-nephritic variety, is distinguished by vascular and renal derangement. The third or neurotrophic form manifests itself mainly by nervous symptoms. Each of these forms necessitates a different mode of treatment. Salicylates and colchicum should be given with caution when there are paroxysmal attacks in the joints. Colchicum is useful when given in small doses if there be no articular manifestations. When the digestive variety is present flesh meat should be forbidden, alkaline drugs given before, and dilute hydrochloric acid after a meal. If there be hepatic or pancreatic insufficiency, calomel or pancreatic preparations should be given respectively. Certain patients are benefited by resort to a thermal "cure" such as Vichy or Chatel-Guyon. The neurotrophic form necessitates severe hygienic treatment intellectually, morally, and physically; hydrotherapy, and a change of air, particularly to mountain air, are useful. This type of patient is generally of gouty parentage, and his life should be free from fatigue and irregularities of diet and regimen.

CONSTIPATION is one of the most common, and at the same time most troublesome, derangements of the system to which the civilised human animal is subject. Any practical suggestions, therefore, for the treatment of this condition without resort to purgatives are especially welcome to the physician. Professor Schmidt, of Dresden, has made a special study of the subject, and on the ground of certain theoretical reasons in regard to the nature and cause of constipation has conceived the idea of administering gelatin to combat the condition. This author attributes constipation in a large number of cases to a threefold cause. In the first place the food is too completely digested and absorbed and leaves a minimum of residue, so that the intestine is not sufficiently distended mechanically for the peristaltic contractions to have their full effect. In the second place, in consequence of the complete and prolonged process of absorption, the intestinal contents are dehydrated too far, so that the fæces are rendered hard and dry, and therefore difficult of expulsion. Lastly, this dehydration hinders the normal processes of fermentation which appear to perform the task of exciting peristalsis, and consequently evacuation. The treatment of such a condition should therefore have a threefold object: increase the volume of the fæces, hydrate them and re-establish fermentation, or, at any rate, the normal peristalsis. Schmidt claims to obtain these results by the use of gelatinous or mucilaginous substances; and he uses especially gelatin or agar-agar, obtained from the seaweed fucus spinosus. It retains the moisture of the intestinal juices, swells, and so enlarges the intestinal contents, and at the same time acts as a sort of lubricant by its soft and unctuous qualities. It may be taken in the form of a fine powder in doses of two to five grammes a day, or it may be combined, if necessary, with some mild laxative such as cascara or senna. Professor Schmidt recommends some such combination at the commencement of the treatment, as taken alone the gelatin may take some days at first before it is effective, and this is likely to discourage the patient and discredit the method. Later, in a large proportion of cases, the gelatin is found to be effective by itself without the admixture of any laxative drug.

ANKYLOSTOMIASIS is attracting much attention from the medical profession in South Africa, and the *Transvaal Medical Journal* publishes two important papers on the question, one from the Witwatersrand, and the other from Natal. The main conclusions arrived at in the former are as follows. Two nematode worms are endemic in certain parts of South Africa, ankylostomum duodenale and necator americanus: the symptoms caused are apparently the same in each case. The high veld and temperate regions generally are fairly free, except for imported cases; whereas the tropical and subtropical regions of Portuguese East Africa and Rhodesia are extensively infected. The proportions of natives arriving at the Rand mines from some of the latter districts and harbouring the worms are as high as 50 per cent., 60 per cent., and more. A very curious fact is that in those who have been in the mines some time, there are fewer cases of ankylostomiasis than in the new arrivals. This, in view of the extremely unhygienic habits of the average Kaffir, is surprising, though gratifying, and it is ascribed to several natural conditions, adverse to the growth of the larvæ, which prevail in most of the mines; such as acidity of water, presence of iron salts, low temperature, and dryness of some mines. It seems to be the general opinion that infection by the skin is not rare, though the alimentary canal is also an avenue of ingress. Europeans are seldom found affected, even among the underground staffs, the exceptions having occurred in mines which differ from the majority in respect of some of the qualities mentioned.

As regards Natal the outlook seems less favourable than in the inland colony. It is but three years since attention was called to the existence of the disease among the Indian coolies who have been imported in large numbers to work the sugar plantations, and it has only recently been discovered that its widespread incidence among them is due to the fact that the great majority of these men are actually infected before they leave India. Unfortunately, a good many cases have occurred among the Kaffir labourers employed on the same estates as the coolies, and not a few Europeans, including children, have also contracted the disease. It would also appear that kala-azar has made entrance into the Garden Colony from the same source, and if this should spread to any extent the people of Natal will have to pay heavy penalties for their attempt to develop the natural resources of their country by the introduction of Asiatic labour.

At the last Congress of the learned societies of the Sorbonne, Dr. Jean Poisson read a paper on the pathogenesis and medico-legal aspect of lumbago. The ætiology of the disease is attributed to either the arthritic or rheumatic diathesis. The following is the author's definition of the disease: "A sudden acute pain of diathetic origin, or due to indirect traumatism, located in the tissues or articulations of the lumbar region without any lesion of the medullary canal or breach of surface of the skin." From the point of view of compensation the question of lumbago is interesting. Workmen often attribute a sudden pain to an accident which is in reality due to lumbago, a disease which insurance companies refuse to admit as accidental. For compensation to be given there must be evidence of some muscular injury. The author has been in the habit of using a mixture of stovain 1 centigram, sodium salicylate 2 centigrams, distilled water 1 c.c., injected sub cutem. In the worst cases three injections will suffice to relieve the patient for 24 hours. The analgesic effect is felt in a few minutes and the patient is able to betake himself home on foot. A traumatic case needs more injections than one, due to the rheumatic or arthritic diathesis, the former needing injections for as much as five days. Massage and purgation are useful. The author has been able to distinguish between traumatic and diathetic cases, the latter returning for further injections when any subsequent attack of pain came on.

A RECENT number of *Il Morgagni* contains a paper by Dr. Mozzi, in which he describes an organism which he has constantly found in the sputum of over sixty children suffering from whooping cough. The organism is a diplococcus formed by the union of two spherical elements. These elements are occasionally somewhat elongated. They are enveloped by an oval capsule. Occasionally the diplococci form chains of six or eight pairs, a capsule then seeming to enclose the whole chain. The cocci stain well with fuchsine. In one case the author found the same organism in the discharge from the ear of a patient who was attacked by acute purulent otitis media, subsequently to the whooping cough. He is of opinion that his diplococcus is the specific organism of the disease.

At a recent meeting of the Académie des Sciences, MM. Rodet and Delanoë have reported upon researches carried out by them with a view to determine the relation between the virulence of the bacillus tuberculosis and the course of pulmonary disease in individual cases. For this purpose cultures were taken from the sputa of twenty-eight cases of pulmonary tuberculosis of the most varied types, from the most acute to the most prolonged and chronic forms and tested by injections into guinea-pigs and rabbits. The virulence of the cultures for these animals was found to vary considerably for the intermediate degrees, but there was almost absolute parallelism in that of the same cultures for the two animals in the extreme cases. There was also a definite relation between the virulence of the bacilli, as determined experimentally in this way and the course of the disease in the patients from whom they were derived, especially in regard to the extreme cases. The authors conclude from this that the great differences which exist in the development of tuberculosis in different individuals depend not so much upon variations in the patients' power of resistance but rather upon the virulence of the infecting bacillus.

ABOUT a year ago Löwi claimed to have discovered a means of detecting affections of the pancreas, the instillation of adrenalin into the conjunctival sac causing a mydriasis in all such cases. More recently Gandola Quadrio has instituted a series of observations at the Medical Clinic in Rome, with the view of testing the diagnostic value of this procedure. Twenty-five persons were submitted to the test, and of these twenty showed no sign of mydriasis after the instillation of the adrenalin, and in none of them was there the least suggestion of pancreatic disturbance. The remaining five gave a positive reaction, and of these four were subjects of pancreatic trouble (two were diabetics and two had pancreatic tumours); the fifth was the subject of epileptic fits, but showed no sign of a pancreatic affection. It seems, therefore, that the method deserves further study on a more extended scale, especially as it is extremely simple and quite harmless. Three or four drops of an adrenalin solution are instilled into the conjunctival sac, and in cases of a positive reaction a definite dilatation of the pupil takes place in the course of a few minutes. The author has never experienced any untoward accident as a result of the instillation. The patients may complain of a slight smarting, which, however, rapidly disappears. No satisfactory theory appears to offer itself at present to account for the connection between the reaction and the functions of the pancreas.

